# A Carabrane-Type Sesquiterpenolide Carabrone from *Carpesium cernuum* Inhibits SW1990 Pancreatic Cancer Cells by Inducing Ferroptosis

**DOI:** 10.3390/molecules27185841

**Published:** 2022-09-09

**Authors:** Yi-Dan Zheng, Ying Zhang, Jun-Yi Ma, Chun-Yan Sang, Jun-Li Yang

**Affiliations:** 1 College of Life Science, Northwest Normal University, Lanzhou 730070, China; 2 CAS Key Laboratory of Chemistry of Northwestern Plant Resources and Key Laboratory for Natural Medicine of Gansu Province, Lanzhou Institute of Chemical Physics, Chinese Academy of Sciences, Lanzhou 730000, China; 3 University of Chinese Academy of Sciences, Beijing 100049, China

**Keywords:** natural product, *Carpesium cernuum* L., SW1990 cells, proteomics, ferroptosis

## Abstract

Pancreatic cancer has an extremely poor prognosis, and the clinical drugs for the treatment of pancreatic cancer are usually multi-drug combinations. Therefore, it is necessary to search for and find specific new bioactive agents against pancreatic cancer. Carabrone is a carabrane-type sesquiterpenolide extracted from *Carpesium cernuum* L., and this natural compound has been reported to be a potential anti-tumor agent. However, there are few reports on the function of carabrone related to anti-tumor activity in pancreatic cancer. Herein, cell experiments indicated that carabrone had anti-proliferation inhibition and anti-migration and anti-invasion activity against SW1990 cells. Furthermore, the tandem mass spectrometry and network pharmacology analysis showed that this activity may be related to the ferroptosis and Hippo signaling pathway. Taken together, our results demonstrated that carabrone exhibited prominent anti-pancreatic cancer activity and could be a promising agent against pancreatic cancer.

## 1. Introduction

*Carpesium cernuum* L. belongs to a perennial herb of the Compositae family. The whole plant of *C. cernuum* is used as a traditional Chinese medicine for clearing away heat and detoxification [1], as well as anti-inflammatory and anti-tumor activities [2]. Its extract has certain cytotoxicity against various human tumor cell lines, such as MDA-MB-231 cells, MCF7 cells, HepG2 cells and A549 cells [3]. In recent years, a variety of biologically active compounds have been isolated and identified from this plant, mainly including sesquiterpenoids [4]. The compound ineupatolide (T-21) isolated from *C. cernuum* has antiproliferative effects against PC-3 human prostate cancer cells by promoting apoptosis and cycle arrest [5]. However, there is no report on the anti-pancreatic cancer of *C. cernuum* and its chemical compounds.

Pancreatic cancer is one of the most common malignant tumors in the digestive tract and threatens the life and health of human beings. In recent years, the incidence of pancreatic cancer has been rising [6]. As one of the deadliest malignant tumors in humans, about 80–85% of patients are diagnosed as locally advanced or even metastasized, but there are no effective drugs [7]. It is of easy recurrence and metastasis because of its insidious onset and the difficulty of early diagnosis. The current 5-year survival rate for pancreatic cancer is only 9%, with only 20% of patients surviving more than 1 year after initial diagnosis [8,9,10]. Therefore, the drug development for pancreatic cancer is still a problem that needs to be overcame, and the research for new anti-cancer drugs is imperative.

Ferroptosis is a newly discovered iron-dependent non-apoptotic cell death method in recent years [11], which is closely related to many diseases, including cancer. The currently recognized ferroptosis-inducing signal is the production of reactive oxygen species (ROS) for various reasons [12] including lipid metabolism, iron metabolism and glutathione metabolism [13]. Deletion of some signaling pathways, such as effector transcriptional regulator 1 (TAZ) in the Hippo signaling pathway, confers ferroptosis resistance in cells; whereas overexpression of TAZS89A sensitizes cells and leads to ferroptosis [14]. Various studies have shown that induction of ferroptosis is an effective modality for pancreatic cancer treatment [15,16]. Natural products are rich in substances and exhibit good biocompatibility and low toxicity to the human body. They play a preventive role in the early stage of tumorigenesis and have a therapeutic role in the tumorigenesis process [17,18]. For instance, piperlongumine [19] and artesunate [20] could induce ferroptosis in PANC-1 cells. In summary, *C. cernuum* is expected to become an important source of active compounds for the development of new drugs to treat pancreatic cancer.

In this study, the cytotoxicity of carabrone from *C. cernuum* was evaluated against four pancreatic cancer cells, including SW1990, CFPAC-1, Capan-2 and PANC-1, and the SW1990 cells were the most susceptible based on the IC_50_ value. The effects of carabrone on the in vitro viability, cell morphology, colony formation, cell migration and MMP-related proteins of SW1990 cells were then examined. In addition, the differentially expressed proteins of carabrone-treated SW1990 cells were identified and analyzed by TMT-based proteomics technology combined with bioinformatics methods. Finally, ROS and mitochondrial membrane potential (MMP) were further analyzed to validate the proteomics results. The underlying mechanism of carabrone against SW1990 could be related with the ferroptosis and Hippo signaling pathway.

## 2. Results

### 2.1. Effects of Carabrone on Viability and Migration in SW1990 Cells

**Figure 1:**
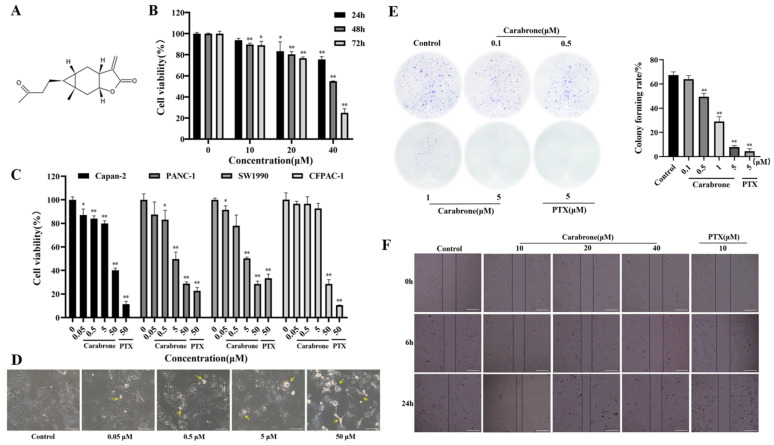
Effect of carabrone on cell viability, proliferation, morphology and migration in SW1990 cells. (**A**) Chemical structure of carabrone. (**B**,**C**) SW1990, CFPAC-1, Capan-2 and PANC-1 cells were evaluated with carabrone to detect cell viability by MTT assay. (**D**) Observation of cell morphology of SW1990 cells treated with carabrone. Damaged cells are indicated by yellow arrows. (**E**) Image of colony formation. Colony forming rate = (number of colonies/number of cells inoculated) × 100% (**F**) Effect of carabrone on wound healing assay of SW1990 cells. Data are presented as mean ± SD (*n* = 3). * *p* < 0.05, ** *p* < 0.01, compared with the control.

To evaluate the cytotoxicity of carabrone against pancreatic cancer, four cell lines (SW1990, CFPAC-1, Capan-2 and PANC-1) were selected. The chemical structure of carabrone is shown in Figure 1A. The four cell lines were treated with different concentrations of carabrone and cell viability was determined. As shown in Figure 1C, carabrone showed cytotoxic activity on the four human pancreatic cancer cell lines, and the IC_50_ values of SW1990, CFPAC-1, Capan-2 and PANC-1 were 5.53 ± 1.19, 48.72 ± 2.90, 47.62 ± 1.72 and 7.78 ± 2.62 μM, respectively. In particular, concentration-dependent inhibition could be observed in 0.05, 0.5, 5 and 50 μM treatments. Based on the IC_50_ value, carabrone had much more inhibitory effect on the proliferation of SW1990 cells. To further study the effect of carabrone on the viability of SW1990 cells, as shown in Figure 1B, carabrone had clear concentration and time dependent inhibitory activity in 10, 20 and 40 μM treatments for 24, 48 and 72 h. The morphology of carabrone-treated SW1990 cells may be disrupted. In the control group, the cells adhered well, existed in sheets, and the morphology was complete. After carabrone treatment, the cells became brighter, could not maintain their original morphology and decreased in number. With the increase of the administered concentration, the cell state became worse, and the intercellular space became dirty (Figure 1D). Based on the above experimental results, SW1990 cells were chosen for subsequent studies.

Colony formation assay indirectly reflected the effect of carabrone-treated SW1990 cells in proliferation and population-dependence. After carabrone treatment of SW1990, the number of formed colonies experienced a great reduction, and the colony forming rate also exhibited a significant reduction in a concentration-dependent manner. Notably, the colony forming rate in SW1990 cells treated with carabrone was comparable to that of paclitaxel (PTX) treatment. Compared with the control, the colony forming rate of the drug treatment group could be decreased by about 7 times at most (Figure 1E). It is indicated that carabrone had good anti-proliferation ability of SW1990 cells. Furthermore, it is found that carabrone significantly suppressed the migration of SW1990 cells. Especially, according to percent wound closure = (wound area at 0 h − wound area at 24 h)/wound area at 0 h × 100%, we also observed a two-to-four-fold decrease in the migratory capacity of SW1990 cells by treatment of cells with carabrone (Figure 1F).

### 2.2. Effects of Carabrone on MMP2 and MMP9 Proteins in SW1990 Cells

**Figure 2:**
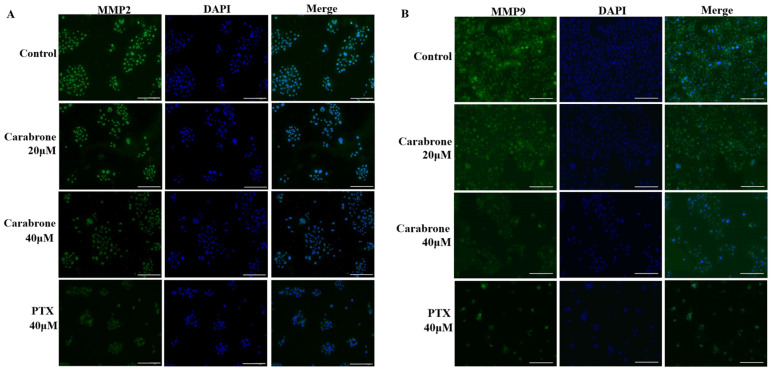
Effects of carabrone on MMP2 and MMP9 proteins in SW1990 cells. (**A**,**B**) Representative fluorescent images of SW1990 cells treated with carabrone and PTX for 48 h. (Scale bars: 100 µm).

Previous wound healing experiments showed that carabrone inhibited the migration of SW1990 cells. Generally, MMP2 and MMP9 proteins are closely related to the development of tumors. Consistently, the invasive capacity of SW1990 cells was substantially diminished with increasing concentration of carabrone. In addition, it is found that different concentrations of carabrone-treated cells inhibited MMP2 and MMP9 proteins (Figure 2A,B). These results further indicated that carabrone inhibited the expression of MMP2 and MMP9 proteins in SW1990 cells, presumably related to its effect on migration and invasion of pancreatic cancer cells. Among them, 40 μM carabrone had the greatest effect on the expression of MMP-related protein in SW1990 cells (Figure 2).

### 2.3. Proteomic Comparison of Carabrone-Treated SW1990 Cells with Control SW1990 Cells

**Figure 3:**
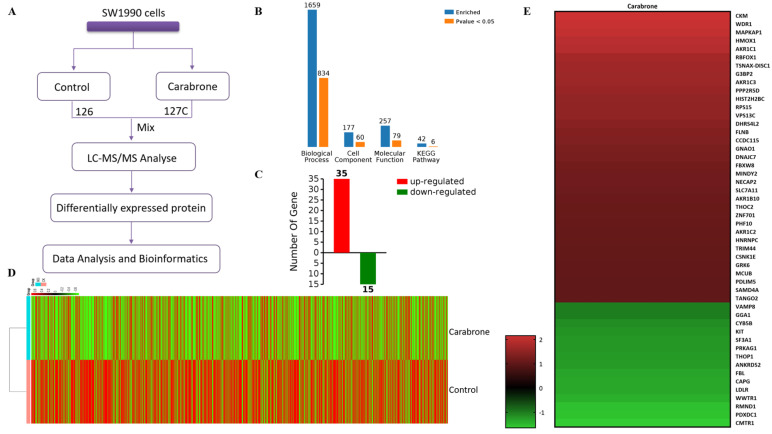
TMT-based proteomics analysis. (**A**) Flowchart for proteomics analysis. Proteins in carabrone-treated SW1990 cells were labeled with 126 and 127C TMT labels. Labeled samples were analyzed by LC-MS/MS and differentially expressed proteins were analyzed by database searching. (**B**) The differentially expressed proteins were enriched and analyzed. (**C**) Number of differentially expressed proteins. (**D**) Heatmap of the expression levels of 9864 total proteins. The red-colored clusters represent up-regulated proteins, and the green-colored clusters represent down-regulated proteins. (**E**) Heatmap of the expression levels of 50 differentially expressed proteins. The red-colored clusters represent up-regulated genes, and the green-colored clusters represent down-regulated genes.

Proteins were extracted from cells treated with carabrone (40 μM). The samples were labeled using 126 and 127C TMT tags, and the labeled protein solution was fractionated into peptides of different sizes which were utilized for LC-MS/MS analysis (Figure 3A). Using LC-MS/MS detection and Proteome Discoverer 2.5, totals of 76,776 peptides and 9864 proteins were identified in the samples. Subsequently, cluster analysis was performed to obtain a heatmap containing 9864 proteins (Figure 3D). Futhermore, a total of 50 differentially expressed proteins was screened based on the *p*-values (*p* < 0.05) and fold change values (FC ≥ 2 or FC ≤ 1/2). Finally, 35 up-regulated (with FC ≥ 2.0) and 15 down-regulated proteins (with FC ≤ 1/2) were identified in the carabrone-treated cell samples. (Figure 3C,E).

### 2.4. Functional Enrichment and Validation of Carabrone Regulatory Proteins

**Figure 4:**
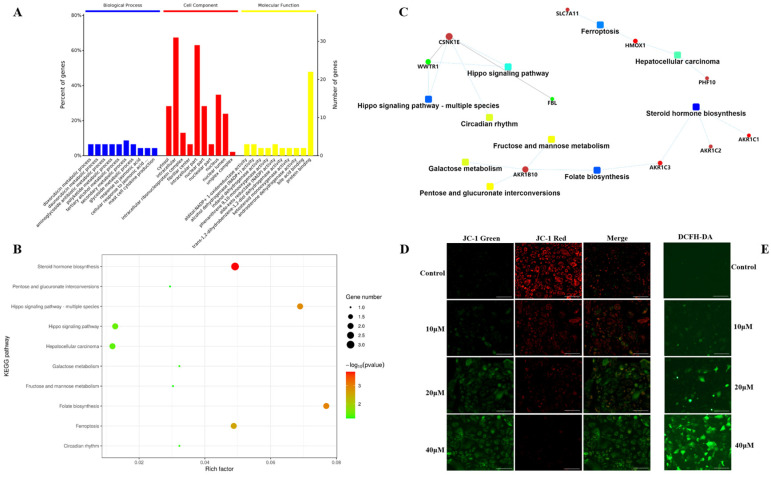
Functional enrichment and validation of carabrone regulatory proteins. (**A**) GO enrichment analysis. (**B**) KEGG enrichment analysis, shown as a pathway bubble chart of the top 10 results of differential proteins. (**C**) PPI network analysis. Circles refer to Protein/Gene and rectangles refer to GO/KEGG terms. (**D**,**E**) Representative fluorescent images of ROS and MMP in SW1990 cells treated with different concentrations of carabrone. (Scale bars: 100 µm).

The obtained protein data were analyzed using bioinformatics methods to get relevant information about the involved pathways. Based on Gene Ontology (GO) analysis, the enrichment of carabrone-related proteins in Biological Processes (BP), Cellular Components (CC) and Molecular Functions (MF) were shown in Figure 3B. In the BP analysis, a total of 1659 BPs were enriched, of which 834 were statistically significant. The majority of identified proteins were relative to metabolic, particularly secondary metabolic and glycoside metabolic processes. In the CC analysis, a total of 177 CCs were enriched in this analysis, of which 60 were statistically significant. The CC analysis showed that most of the identified proteins belonged to the intracellular space and the nucleus. In the MF analysis, a total of 257 MFs were enriched, of which 79 were statistically significant. The molecular functional classification revealed that most of these proteins were involved in protein binding, alcohol dehydrogenase (NADP+) activity and aldo-keto reductase (NADP) activity (Figure 4A). Based on the above GO analysis, it is concluded that these differentially expressed proteins in carabrone-treated SW1990 cells have a wide range of biological processes, cell distribution and functions, which is consistent with reports that carabrone has multiple pharmacological activities. Moreover, a total of 42 pathways were enriched in the Kyoto Encyclopedia of Genes and Genomes (KEGG) analysis, of which 6 pathways were statistically significant (Figure 2B). The top 6 pathways, including steroid hormone biosynthesis (hsa00140), folate biosynthesis (hsa00790), Hippo signaling pathway-multiple species (hsa04392), ferroptosis (hsa04216), Hippo signaling pathway (hsa04390), and hepatocellular carcinoma (hsa05225). Among them, the steroid hormone biosynthesis was the most significant and enriched pathway with the largest number of proteins (Figure 4B).

Moreover, the (Protein–Protein Interaction) PPI network was analyzed to further explore the all-sided information from the identified protein data. The network model was generated using the Omics Bean web application based on information gained in up to 5 levels of functional analysis: fold-change of protein/gene expression, GO/KEGG term enrichment, transcription enrichment, micro RNA enrichment and metabolite enrichment. The PPI analysis identified the steroid hormone biosynthesis as the most significantly enriched pathways. A merged network is shown in Figure 4C that the proteins indicated by red circle nodes were up-regulated while those indicated by green were down-regulated. Most proteins were down-regulated in ferroptosis, hepatocellular carcinoma, steroid hormone biosynthesis, folate biosynthesis, fructose and mannose metabolism, galactose metabolism and circadian rhythm, whereas dysregulated proteins in the Hippo signaling pathway were up-regulated and down-regulated.

Based on the above proteomics analysis, the abnormal expression of SLC7A11 and HO-1 indicated that ferroptosis might be the main cause of carabrone against SW1990 cells. Ferroptosis is a specific ROS-dependent death, with lipid peroxidation as the central link [21,22] and is morphologically characterized by mitochondrial damage [23,24]. As shown in Figure 4E, the fluorescence intensity of SW1990 cells was observed qualitatively by using DCFH-DA staining. Compared with the control group, the fluorescence intensity increased gradually with the increase of the concentration, which showed that ROS was increased in SW1990 cells treated with carabrone. Meanwhile, the mitochondrial membrane potential (MMP) changes in SW1990 cells were detected by JC-1 fluorescent probe (Figure 4D). With the gradual increase of carabrone concentration, the green fluorescence was enhanced, and the red fluorescence was weakened, which revealed that carabrone decreased MMP in SW1990 cells. These results indicated that the cells tended to ferroptosis, which was consistent with the proteomics analysis.

## 3. Discussion

Pancreatic cancer is one of the most lethal cancers in humans, and more than 90% belong to pancreatic ductal adenocarcinomas [25]. Surgical excision, chemotherapy and radiotherapy are the main therapeutic options. Surgical excision is currently the only therapy that can be curative [26]. Carabrone is a carabrane-type sesquiterpenolide isolated from *C. cernuum*, and this compound has been reported to show multiple antitumor activities [27]. Previous studies showed that carabrone has certain cytotoxicity to liver, melanoma, breast and oral epidermoid cancer cells [27,28,29]. However, to the best of our knowledge, there is not any report on the proliferation inhibition on pancreatic cancer cells by carabrone.

In this paper, the bioactivity and the mechanism of carabrone inhibiting SW1990 cells was discussed. First, we investigated the cytotoxicity of carabrone against four pancreatic cancer cell lines, SW1990, Capan-2, CFPAC-1 and PANC-1 cells. According to the IC_50_ values, carabrone was shown to have the most susceptibility in SW1990 cells, which were selected for further research. Carabrone significantly inhibited cell proliferation in a time-dependent and concentration-dependent manner, interfering with colony formation and migration. MMP2 and MMP9 proteins could rapidly hydrolyze the components of the intercellular matrix and the extracellular basement membrane, and they could destroy the natural barrier of tumor metastasis [30]. Immunofluorescence staining showed that the MMP2 and MMP9 proteins of SW1990 cells treated with carabrone had a downward trend, compared with the control group, which was comparable to that of the PTX group. Proteomic analysis is a powerful tool for identifying biomarkers and evaluating biological networks [31], its evaluation about understanding of protein interconnections and their overall biological network signaling in pancreatic cancer cells after drug administration is crucial. Secondly, the TMT-based proteomics method was used to analyze the molecular targets of SW1990 cells treated with carabrone. Moreover, protein function annotation and differentially-expressed protein statistical analysis was performed. In all, proteomics provided a new perspective for elucidating carabrone as a potential drug lead for pancreatic cancer. Based on the above findings, 50 differentially-expressed proteins were observed in SW1990 cells treated with carabrone, of which 35 were up-regulated and 15 were down-regulated (Tables S1 and S2, Supplementary Materials). These differentially expressed proteins were analyzed by GO annotation, and they were enriched in three aspects: BP, CC and MF. Data from multiple clinical trials showed that the expression of iron metabolism-related proteins was positively correlated with the size of solid tumors in pancreatic cancer [32]. Essentially, there is an abnormally high level of iron in pancreatic cancer cells, which played an important role in the occurrence and development of tumor cells [32]. Ferroptosis is a new form of cell death that is completely different from apoptosis, and its central link is an accumulation of iron-mediated lipid peroxidation [33,34]. This study showed that, notably, HO-1, SLC7A11 and MAPKAP1 were the three proteins that were up-regulated in SW1990 cells after carabrone treatment (Figure S1). Heme oxygenase is the main source of intracellular iron [35]. Among them, HO-1 can antagonize oxidative stress and is also mainly associated with ferroptosis and degrades heme and releases Fe^2+^, as well as generating a large amount of ROS via the Fenton reaction [35]. For instance, Kwon showed that HO-1 can promote Erastin-mediated ferroptosis in HT-1080 fibrosarcoma cells [36]. We then turned our attention to SLC7A11, which is the catalytic subunit of System Xc^−^. When System Xc^−^ and cystine absorption were inhibited, ferroptosis occurs. Among these, SLC7A11 was compensatorily transcriptionally upregulated [37]. Thus, inhibition of SLC7A11 also promoted the occurrence of ferroptosis. MAPKAP1 is a subunit of mTORC2, which can inhibit the transporter activity of SLC7A11 by phosphorylating SLC7A11 [38]. Since the down-regulation of GSH expression mediates cellular lipid peroxidation, negative feedback leads to the up-regulation of ROS, which also leads to the occurrence of ferroptosis [39,40]. In the presence of ferroptosis, mitochondria of cells were damaged and resulted in an MMP decrease [41]. WWTR1 is a transcriptional coactivator of downstream regulatory targets of the Hippo signaling pathway and plays a key role in tumors by limiting proliferation and promoting apoptosis [42,43]. In this study, CSNK1E was up-regulated and WWTR1 was down-regulated in SW1990 cells treated with carabrone, thereby regulating the Hippo pathway, which was consistent with the experimental results that cell proliferation was inhibited and cell wounds no longer healed after treatment (Figure S2).

**Figure 5:**
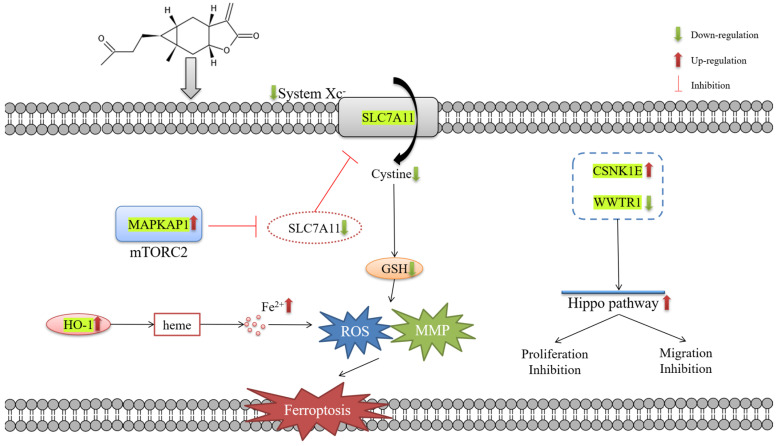
Schematic figure of the cancer inhibition mechanism of carabrone in pancreatic cancer.

In conclusion, it is highly probable that carabrone showed cytotoxicity against SW1990 pancreatic cancer cells through proliferation and migration inhibition mediating via the Hippo signaling pathway and finally induced the cell death through ferroptosis (Figure 5).

## 4. Materials and Methods

### 4.1. Chemicals and Reagents

Carabrone was received from the CAS Key Laboratory of Chemistry of Northwestern Plant Resources, and the HPLC purity level was >95%. Paclitaxel (PTX, purity = 99.96%) was purchased from MedChemExpress.

### 4.2. Cell Culture

Four human pancreatic cancer lines (SW1990, CFPAC-1, Capan-2 and PANC-1) were procured from the cell bank of the Chinese Academy of Sciences (Shanghai, China). Cells were cultured in Dulbecco’s modified Eagle’s medium (DMEM, Gibco, Carlsbad, CA, USA) comprising 10% fetal bovine serum (FBS, Sijing, Hangzhou, China) at 37 °C in a 5% CO_2_ incubator.

### 4.3. Cell Viability Assay

MTT (Solarbio, Beijing, China) assay was performed to determine cell viability. Cells (3 × 10^3^/well) were seeded into 96-well plates and incubated with diverse concentrations of carabrone for 72 h. The old medium was thrown away and 100 μL of medium containing 10 μL of MTT solution was added to each well and further cultured for 4 h. The optical density (OD) of the samples was measured at 490 nm using a Microplate Reader (Rayto, Shenzhen, China).

### 4.4. Wound Healing Assay

SW1990 (1 × 10^6^/well) cells were seeded into 6-well plates, cultured overnight and the medium was removed and rinsed with phosphate-buffered saline (PBS, Solarbio, Beijing, China), scored vertically in 6-well plates with a 100 μL gun tip, washed again with PBS and placed in the incubator; cell migration was observed under the microscope at 0 h, 6 h and 24 h and photographed and recorded. Migration rates were calculated by Image J.

### 4.5. Immunofluorescence

SW1990 cells (1 × 10^5^/well) were seeded into 6-well plates and cultured overnight, and the cells were treated with carabrone for 48 h. Cells were stimulated with 0.3% Triton X-100 (Solarbio, Beijing, China) for 20 min. Subsequently, cells were blocked with Immunostaining Blocking Solution (Beyotime, Shanghai, China) for 1 h and then incubated with the MMP2 primary antibody (proteintech, Chicago, IL, USA) at 4 °C overnight. The next day, cells were incubated with Fluorescein (FITC)-conjugated secondary antibody (proteintech, Chicago, IL, USA) for 1 h at 37 °C, and then visualized after 3 min of DAPI (Solarbio, Beijing, China) staining. Staining was examined using the Mshot inverted fluorescence microscope (Guangzhou, China) at 10× magnification (scale bar 100 μm). The color of MMP2 protein is greenish and the color of nucleus was blueish. Performed a merged picture to show co-location.

### 4.6. Colony Formation Assay

The SW1990 cells (1 × 10^3^/well) were seeded into 6-well plates. Then, cells were treated with carabrone. PTX was used as a positive control. After 15 days, colonies were fixed with 4% paraformaldehyde (Solarbio, Beijing, China) for 20 min and stained with crystal violet (Solarbio, Beijing, China) for 30 min before observation.

### 4.7. Intracellular ROS Detection Analysis

The SW1990 cells (1 × 10^5^/well) were seeded into 6-well plates. Then, cells were treated with carabrone. In each group, intracellular ROS levels were detected using a ROS assay kit (Beyotime, Shanghai, China) according to the manufacturer’s instructions. Then, cells were photographed under a fluorescence microscope.

### 4.8. Protein Preparation and TMT Labeling

Cells were treated with 40 μM of carabrone for 24 h. RIPA Lysis was added, and protein concentration was determined by Pierce™ BCA Protein Assay Kit (Thermo Fisher Scientific, Waltham, MA, USA). The 20 μL of TMT labeling reagent was added to the sample, and the reaction was incubated for 1 h at room temperature. Subsequently, 1 μL of 5% hydroxylamine was added and incubated for 15 min to quench the reaction. The column was placed into a new 2.0 mL sample tube, in which 300 μL of 5% ACN (Fisher Chemical, Waltham, MA, USA) and 0.1% TEA were loaded to remove unreacted TMT reagent. Finally, each sample was evaporated to dryness using vacuum centrifugation.

### 4.9. Nano LC-MS/MS Analysis

The tryptic peptides were dissolved in solvent A (0.1% formic acid in water) and directly loaded onto a chromatographic column RPLC C18 (1.9 μm particles, 150 μm × 15 cm), all at a constant flow rate of 600 nL/min on an EASY-nLC 1200 UPLC system (ThermoFisher Scientific, Waltham, MA, USA).

The peptides were subjected to an ESI nanospray source followed by tandem mass spectrometry (MS/MS) in Orbitrap Eclipse (Thermo) coupled online to the UPLC. The electrospray voltage applied was 2.2 kV. The *m*/*z* scan range was 350 to 1500 for a full scan, and intact peptides were detected in the Orbitrap (Thermo Fisher Scientific, Waltham, MA, USA) at a resolution of 60,000. Peptides were then selected for MS/MS using the NCE setting as 36, and the fragments were detected in the Orbitrap at a resolution of 17,500. The data-dependent procedure that we performed alternated between one MS scan followed by 20 MS/MS scans with a 54 ms dynamic exclusion. Automatic gain control (AGC) was set at 25E4. The fixed first mass was set to 100 *m*/*z*.

### 4.10. Bioinformatics Analysis and Data Analysis

The raw MS files were analyzed and searched against uniprot-Homo sapiens protein database based on the species of the samples using Proteome Discoverer 2.5. Only high confident identified peptides were chosen for downstream protein identification analysis. Omicsbean (http://www.omicsbean.cn, accessed on 10 February 2022) was used to analyze the obtained proteomic data.

### 4.11. Statistical Analysis

Data were expressed as the means ± SDs based on at least 3 independent experiments. Proteomic data statistical differences were determined via one-way analysis of variance using SPSS 25.0. Other data statistical differences were performed with Student’s *t*-test or one-way ANOVA using GraphPad Prism 8, and values of *p* < 0.05 were considered significant.

## 5. Conclusions

In this paper, we investigated the proliferation inhibition and migration inhibition of carabrone in SW1990 cells and also revealed the mechanism of its anti-tumor activity. Proteomics analysis showed that carabrone inhibited the proliferation and migration of SW1990 cells by up-regulating CSNK1E and down-regulating WWTR1 to activate the Hippo signaling pathway. Most notably, carabrone ultimately led to ferroptosis by down-regulating SLC7A11 and up-regulating HO-1.

## Data Availability

Data are contained within the article.

## Supplementary Materials

The following supporting information can be downloaded at: https://www.mdpi.com/article/10.3390/molecules27185841/s1, Figure S1: KEGG pathway enrichment map of ferroptosis; Figure S2: KEGG pathway enrichment map of Hippo signaling pathway; Table S1: List of up-regulated proteins in carabrone-treated SW1990 cells; Table S2: List of down-regulated proteins in carabrone-treated SW1990 cells.

## Sample Availability

Samples of the compounds are not available from the authors.
