# Supplementary material for: A Carabrane-Type Sesquiterpenolide Carabrone from Carpesium cernuum Inhibits SW1990 Pancreatic Cancer Cells by Inducing Ferroptosis
Source: Molecules. 2022 Sep 9;27(18):5841. doi: 10.3390/molecules27185841 (PMC9503519; doi:10.3390/molecules27185841)
Supplement: Supplementary file 1 [file molecules-27-05841-s001.zip › molecules-1886096-supplementary.pdf]

Supplementary Materials:

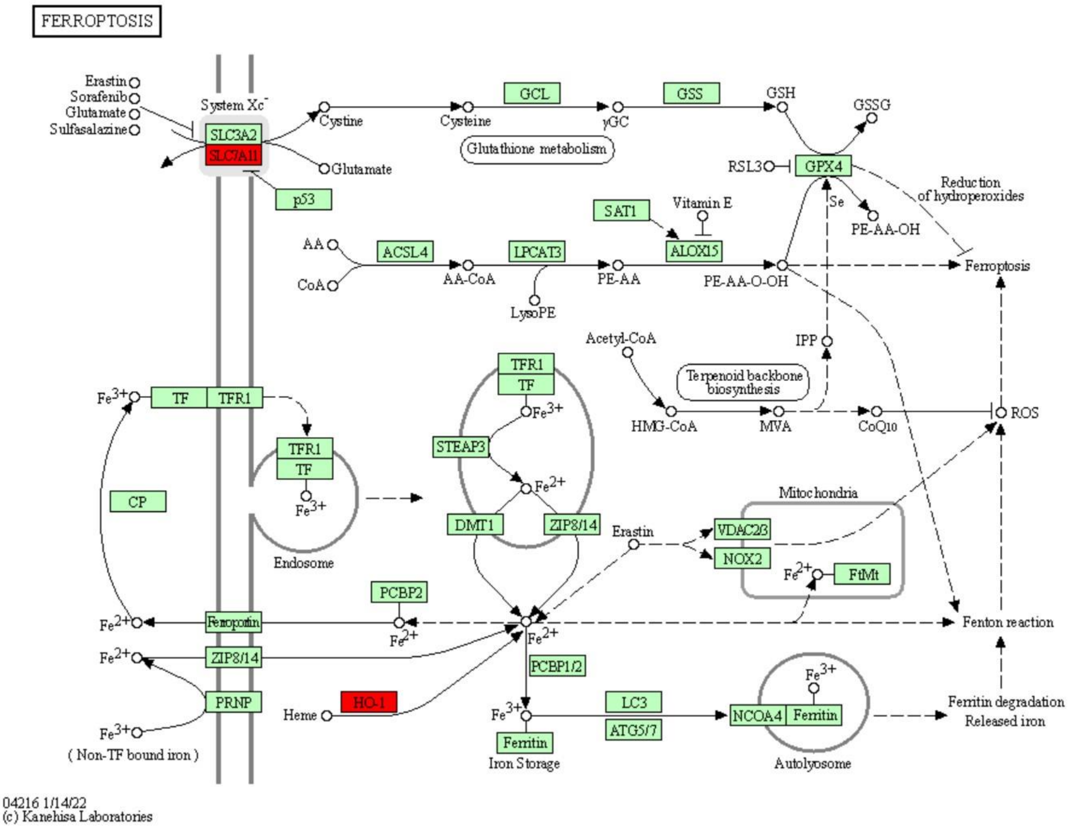

Figure S1 KEGG pathway enrichment map of ferroptosis

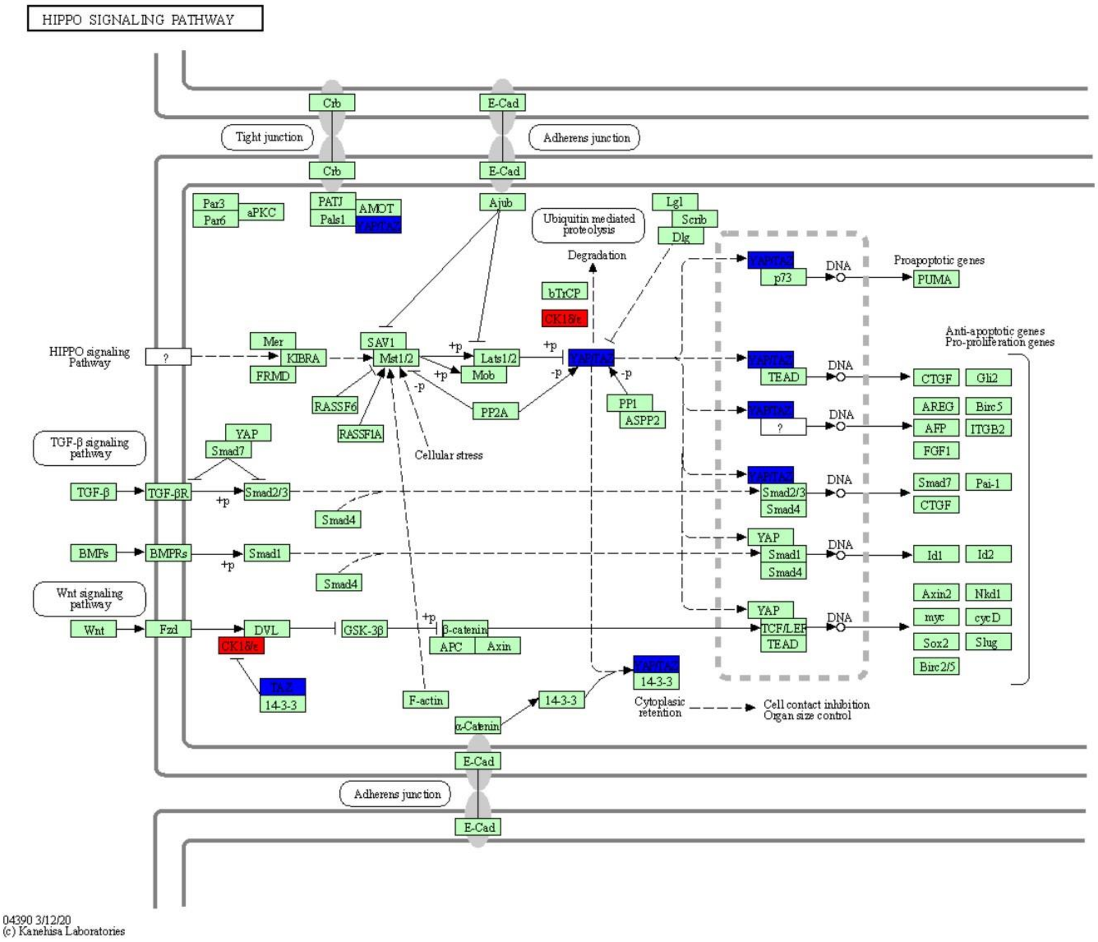

Figure S2 KEGG pathway enrichment map of Hippo signaling pathway

Table S1. List of up-regulated proteins in Carabrone-treated SW1990 cells

ID(Uniprot)	Gene name	Protein name	Fold change
P06732	CKM	Creatine kinase M-type	4.46413809
D6RD66	WDR1	WD repeat-containing protein 1	4.277478742
Q9BPZ7	MAPKAP1	Target of rapamycin complex 2 subunit MAPKAP1	4.111985769
P09601	HMOX1	Heme oxygenase 1	3.853297642
Q04828	AKR1C1	Aldo-keto reductase family 1 member C1	3.758072809
B7Z1U7	RBFOX1	RNA binding protein fox-1 homolog 1	3.309064847
C4P0D6	TSNAX-DISC1	Disrupted in schizophrenia 1 isoform 49	3.184301416
A0A7I2V547	G3BP2	Ras GTPase-activating protein-binding protein 2	3.145660325
A0A0A0MSS8	AKR1C3	Aldo-keto reductase family 1 member C3	3.127165734
H0Y8C4	PPP2R5D	Serine/threonine-protein phosphatase 2A 56 kDa regulatory subunit delta isoform	3.006482812
Q6DN03	HIST2H2BC	Putative histone H2B type 2-C	2.866081285
K7EJ78	RPS15	40S ribosomal protein S15	2.851994653
A0A3B3IT88	VPS13C	Vacuolar protein sorting-associated protein 13C	2.850671896

A0A087WXI8	DHRS4L2	Dehydrogenase/reductase SDR family member 4-like 2	2.678490778
A0A0C4DGA1	FLNB	Filamin-B	2.574865739
B8ZZ99	CCDC115	Coiled-coil domain-containing protein 115	2.555768696
A0A1W2PS82	GNAO1	Guanine nucleotide-binding protein G(o) subunit alpha	2.450882929
A0A6I8PR93	DNAJC7	DnaJ homolog subfamily C member 7	2.412930147
Q8N3Y1	FBXW8	F-box/WD repeat-containing protein 8	2.289470046
H0YM15	MINDY2	Ubiquitin carboxyl-terminal hydrolase	2.25952228
F6SKB8	NECAP2	Adaptin ear-binding coat-associated protein 2	2.250370084
Q9UPY5	SLC7A11	Cystine/glutamate transporter	2.229452467
O60218	AKR1B10	Aldo-keto reductase family 1 member B10	2.163554532
A0A0C4DG98	THOC2	THO complex subunit 2	2.134323943
Q9NV72	ZNF701	Zinc finger protein 701	2.130209544
Q8WUB8	PHF10	PHD finger protein 10	2.107242765
P52895	AKR1C2	Aldo-keto reductase family 1 member C2	2.09806458
B4DY08	HNRNPC	Heterogeneous nuclear ribonucleoproteins C1/C2	2.07673878
Q96DX7	TRIM44	Tripartite motif-containing protein 44	2.072193307
A0A669KBC0	CSNK1E	Casein kinase I isoform epsilon	2.06392984
P43250	GRK6	G protein-coupled receptor kinase 6	2.04333173
Q9NWR8	MCUB	Calcium uniporter regulatory subunit MCUb, mitochondrial	2.030545361
A0A0D9SFW3	PDLIM5	PDZ and LIM domain protein 5	2.028474838
G3V2R1	SAMD4A	Protein Smaug homolog 1	2.023389279
A0A0A0MSI5	TANGO2	Transport and Golgi organization protein 2 homolog	2.018132774

Table S2. List of down-regulated proteins in Carabrone-treated SW1990 cells

ID(Uniprot)	Gene name	Protein name	Fold change
C9JXZ5	VAMP8	Vesicle-associated membrane protein 8	0.499106805
B0QYS5	GGA1	ADP-ribosylation factor-binding protein GGA1	0.498233524
O43169	CYB5B	Cytochrome b5 type B	0.457476096
P10721	KIT	Mast/stem cell growth factor receptor Kit	0.441453017
H7C1L2	SF3A1	Splicing factor 3A subunit 1	0.432677643
H0YIC9	PRKAG1	5'-AMP-activated protein kinase subunit gamma-1	0.432483046
K7EP46	THOP1	Thimet oligopeptidase	0.424173777
Q8NB46	ANKRD52	Serine/threonine-protein phosphatase 6 regulatory ankyrin repeat subunit C	0.420355522
M0R2U2	FBL	rRNA 2'-O-methyltransferase fibrillarin	0.402679073
H7C0X8	CAPG	Adseverin	0.395740201
H0YMD1	LDLR	Low-density lipoprotein receptor	0.395676383
Q9GZV5	WWTR1	WW domain-containing transcription regulator protein 1	0.376390821
A0A2R8YFC3	RMND1	Required for meiotic nuclear division protein 1 homolog	0.344551249
H3BNL6	PDXDC1	Pyridoxal-dependent decarboxylase domain-containing protein 1	0.336021875
H0YCQ1	CMTR1	Cap-specific mRNA (nucleoside-2'-O-)-methyltransferase 1	0.320973707
